# Autoantibody-Mediated Erythrophagocytosis Increases Tuberculosis Susceptibility in HIV Patients

**DOI:** 10.1128/mBio.03246-19

**Published:** 2020-02-25

**Authors:** Youchao Dai, Yi Cai, Xin Wang, Jialou Zhu, Xiaoqian Liu, Houming Liu, Linghua Li, Yinze Zhang, Shengze Liu, Zhihua Wen, Carl G. Feng, Xinchun Chen, Xiaoping Tang

**Affiliations:** a Research Institute of Infectious Diseases, Guangzhou Eighth People’s Hospital, Guangzhou Medical University, Guangzhou, China; b Guangdong Key Laboratory of Regional Immunity and Diseases, Department of Pathogen Biology, Shenzhen University School of Medicine, Shenzhen, China; c International Cancer Center, Shenzhen University General Hospital, Shenzhen University Clinical Medical Academy, Shenzhen, China; d Department of Infectious Disease, Shenzhen People’s Hospital, 2nd Clinical Medical College of Jinan University, Shenzhen, Guangdong Province, China; e Integrated Chinese and Western Medicine Postdoctoral Research Station, Jinan University, Guangzhou, Guangdong Province, China; f Clinical Laboratory, Shenzhen Third People’s Hospital, Shenzhen University School of Medicine, Shenzhen, China; g Yuebei Second People’s Hospital, Shaoguan, China; h Immunology and Host Defense Group, Department of Infectious Diseases and Immunology, Faculty of Medicine and Health, The University of Sydney, Sydney, NSW, Australia; Max Planck Institute for Infection Biology

**Keywords:** HIV, erythrophagocytosis, heme oxygenase-1, autophagy, tuberculosis

## Abstract

Macrophage dysfunction is associated with increased tuberculosis (TB) susceptibility in patients with human immunodeficiency virus (HIV) infection. However, the mechanisms underlying how HIV infection impairs macrophage function are unclear. Here, we found that levels of autoantibodies against red blood cells (RBCs) were significantly elevated in patients with HIV as determined by direct antiglobulin test (DAT). DAT positivity was significantly associated with TB incidence in both univariate and multivariate analyses (odds ratio [OR] = 11.96 [confidence interval {CI}, 4.68 to 30.93] and 12.65 [3.33 to 52.75], respectively). *Ex vivo* analysis showed that autoantibodies against RBCs enhanced erythrophagocytosis and thus significantly impaired macrophage bactericidal function against intracellular Mycobacterium tuberculosis. Mechanistically, autoantibody-mediated erythrophagocytosis increased heme oxygenase-1 (HO-1) expression, which inhibited M. tuberculosis-induced autophagy in macrophages. Silencing *ATG5*, a key component for autophagy, completely abrogated the effect of erythrophagocytosis on macrophage bactericidal activity against M. tuberculosis. In conclusion, we have demonstrated that HIV infection increases autoantibody-mediated erythrophagocytosis. This process impairs macrophage bactericidal activity against M. tuberculosis by inhibiting HO-1-associated autophagy. These findings reveal a novel mechanism as to how HIV infection increases TB susceptibility.

## Abstract (precis)

HIV infection significantly increases TB susceptibility due to CD4 T-cell loss and macrophage dysfunction. Although it is relatively clear that CD4 T-cell loss represents a direct effect of HIV infection, the mechanism underlying how HIV infection dampens macrophage function is unknown. Here, we show that HIV infection enhances autoantibody-mediated erythrophagocytosis, which dampens macrophage bactericidal activity against TB by inhibiting HO-1-associated autophagy. Our findings reveal a novel mechanism explaining how HIV infection increases susceptibility to TB. We propose that DAT could be a potential measure to identify HIV patients who are at high TB risk and who would be suitable for anti-TB chemotherapy preventive treatment.

## INTRODUCTION

Tuberculosis (TB) caused by Mycobacterium tuberculosis infection is one of the 10 most common causes of mortality worldwide and the leading cause of mortality from a single infectious agent; ∼10 million new cases were reported in 2017, with 1.6 million deaths (1). Human immunodeficiency virus (HIV) infection is a strong risk factor for disease progression in TB and is thus associated with poor treatment outcomes (2–4). The HIV-mediated depletion of CD4 T cells that typically confers a protective immune response to M. tuberculosis infection is likely a main driver of the increased prevalence of active TB in countries with a high HIV burden (5–7). Interestingly, increased TB risk has also been reported among patients with HIV and normal CD4 T-cell counts (8, 9). Indeed, besides CD4 T-cell loss, macrophage function is altered during HIV infection also (10, 11). Macrophage-driven innate immunity has been increasingly recognized as having a critical role in the host defense against TB (12); fine-tuning of macrophage fate and function is essential to M. tuberculosis infection outcomes (13). However, the mechanisms underlying how HIV infection impairs macrophage-mediated defenses against M. tuberculosis remain to be elucidated.

HIV infection can induce the production of various autoantibodies, which leads to the development of autoimmune diseases (14). It is reported that 20% to 40% of patients with HIV are positive for anti-red blood cell (RBC) autoantibodies, which can be detected using a direct antiglobulin test (DAT) (15, 16). Treatment with heme, a major component of lysed RBCs, triggers macrophage death, with characteristics of programmed necrosis, and inhibits bactericidal activity against M. tuberculosis (17). In addition, stored RBCs for transfusion can suppress the macrophage defense against Pseudomonas aeruginosa infection though elevated circulating heme levels (18). The presence of anti-RBC autoantibodies can sensitize RBCs *in vivo* and trigger accelerated RBC phagocytosis and destruction by macrophages (19, 20). We therefore hypothesized that anti-RBC autoantibodies might impair macrophage functions to fight against TB by enhancing erythrophagocytosis (macrophagic engulfment of RBCs).

To test our hypothesis, first, we investigated the association between the presence of anti-RBC autoantibodies and the increased risk of TB in patients with HIV. Second, we determined the effect and mechanism of erythrophagocytosis enhanced by anti-RBC autoantibodies on macrophage bactericidal activity against M. tuberculosis.

## RESULTS

### Enhanced anti-RBC autoantibody production in patients with HIV is associated with increased risk of development of active TB.

We first aimed to investigate the presence of anti-RBC autoantibodies in patients with HIV and its association with TB susceptibility. DATs were used to detect anti-RBC autoantibodies in 267 HIV-seropositive patients with (*n* = 23, 9%) or without (*n* = 244, 91%) TB. Here, 28/267 cases (10%) were DAT positive, but DAT positivity was significantly higher in HIV patients with TB than in those without TB (48% versus 7%, *P* < 0.001) (Table 1).

**TABLE 1:** Univariate and multivariate logistic regression analysis of factors associated with TB susceptibility in HIV patients^*a*^

Parameter	Value(s)					
	HIV without TB (*n* = 244)	HIV with TB^*b*^ (*n* = 23)	Univariate analysis OR [95% CI]	*P*	Multivariate analysis [95% CI]^*c*^	*P*
No. of males/total no. of patients (%)	185/244 (76)	19/23 (83)	1.39 [0.52–4.59]	0.533		
Yrs of age (range)	40 (32–48)	34 (29–42)	0.95 [0.90–0.99]	**0.008**	0.95 [0.90–1.00]	**0.038**
Log HIV load (range)	4.69 (3.96–5.28)	4.18 (3.97–4.98)	1.07 [0.82–1.49]	0.668		
WBC count at 10^9^/liter (range)	7.90 (6.07–9.20)	9.20 (6.70–10.75)	1.21 [1.02–1.43]	**0.030**	1.16 [0.96–1.40]	0.121
Hemoglobin levels in g/liter (range)	139 (129–152)	138 (115–147)	0.98 [0.96–1.00]	**0.041**	1.03 [0.99–1.08]	0.121
No. of patients with anemia/total no. of patients (%)^*d*^	35/244 (14)	10/23 (44)	4.59 [1.87–11.08]	**0.001**	3.32 [0.54–21.21]	0.193
No. of CD4 T cells/μl (range)^*e*^	357 (198–698)	436 (259–835)	1.00 [1.00–1.00]	0.325		
No. (%) of DAT-positive results	17/244 (7)	11/23 (48)	11.96 [4.68–30.93]	**<0.001**	12.65 [3.33–52.75]	**<0.001**

a TB, tuberculosis; HIV, human immunodeficiency virus; OR, odds ratio; CI, confidence interval; WBC, white blood cell; RBC, red blood cell; DAT, direct antiglobulin test. Categorical variables are expressed as absolute numbers and percentages, whereas continuous variables are reported as medians and interquartile ranges. Significant differences (*P* < 0.1 in univariable analysis and *P* < 0.05 in multivariable analysis) are highlighted in bold.

b TB was diagnosed by experienced specialists if (i) sputum samples were positive for AFB, NAAT, or M. tuberculosis culture (ii) or results showed no sputum or negative smear results but showed high-resolution computed tomography (HRCT) evidence, positive IGRA, and symptoms responding to TB treatment.

c The likelihood ratio test had a *P* value of <0.0001, and The Hosmer and Lemeshow goodness-of-fit (GOF) test had a *P* value = 0.7206. Both tests indicated that the multivariable logistic regression model was a good fit to the data.

d Anemia was diagnosed at hemoglobin levels of <130 g/liter for males and <115 g/liter for females.

e CD4 T-cell data were missing for 19 cases in HIV without TB and three cases in HIV with TB.

To determine the risk of TB in patients with HIV, various clinical parameters with potential to affect the host defense system were obtained and input into a univariate model to calculate the risk odds ratio (OR) (Table 1). In line with a previous report (21), anemia was significantly associated with TB (OR = 4.59 [confidence interval {CI}, 1.87 to 11.08], *P* < 0.001); however, following adjustment for other variables in the multivariate analysis, anemia was not significantly associated with TB incidence (OR = 3.32 [0.54 to 21.21], *P* = 0.121). By comparison, DAT positivity was significantly associated with TB incidence in both univariate and multivariate analyses (OR = 11.96 [4.68 to 30.93] and 12.65 [3.33 to 52.75], respectively; both *P* < 0.001). Therefore, extending previous findings on the association between anemia and DAT (16, 22), our data provide evidence that increased anti-RBC autoantibody production in HIV is associated with increased TB risk.

### Enhanced erythrophagocytosis in patients with HIV is mediated by anti-RBC autoantibodies.

**FIG 1:**
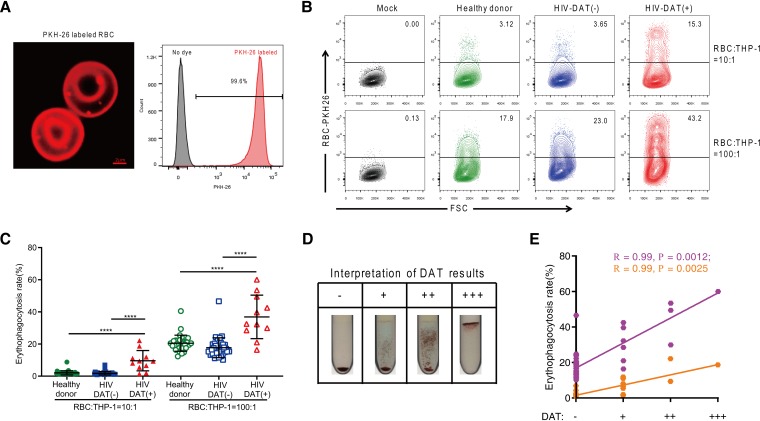
RBCs from patients with HIV with a positive direct antiglobulin test (DAT) result increase erythrophagocytosis by macrophages. (A) RBCs labeled with a membrane-integrating dye (PKH-26) were assessed by confocal fluorescence microscopy (left; bars, 2 μm) and flow cytometry (right; labeling yield, 99.6%). (B and C) *Ex vivo* phagocytosis of PKH-26-labeled RBCs from healthy donors (*n* = 33) and patients with HIV with negative (*n* = 36) and positive (*n* = 11) DAT results upon incubation with PMA-differentiated THP-1 macrophages for 6 h at RBC/macrophage (R/M) ratios of 10:1 and 100:1 revealed by forward scatter (FSC) data (B) and quantified by flow cytometry (C). (D) DAT results were classified as negative (−) or positive (with 1+ [+], 2+ [++], and 3+ [+++] degrees) corresponding to the RBC agglutination strength. (E) Correlation between erythrophagocytosis rate and DAT agglutination level at both R/M ratios (orange, R/M ratio = 10:1; purple, R/M ratio = 100:1), assessed using Spearman’s rank correlation. The data represent means ± standard deviations and are representative of results from at least three independent experiments. ****, *P < *0.0001.

To dissect the causal relationship between anti-RBC autoantibodies and TB susceptibility, we examined macrophage function. Macrophages have critical roles in fighting against M. tuberculosis and clearing senescent or damaged RBCs (12, 23). A positive DAT result indicates RBC sensitization by autoantibodies that trigger accelerated RBC phagocytosis and destruction by macrophages (19, 20). We labeled RBCs isolated from 33 healthy donors and 47 HIV-seropositive patients (including 36 DAT-negative and 11 DAT-positive cases) with a membrane-integrating dye (PKH-26) (Fig. 1A) and determined RBC uptake by phorbol myristate acetate (PMA)-differentiated THP-1 macrophages by flow cytometry (see Table S1 in the supplemental material).

10.1128/mBio.03246-19.8 TABLE S1 Demographics and clinical characteristics of the subjects selected for erythrophagocytosis assay. Download Table S1, DOC file, 0.04 MB. Copyright © 2020 Dai et al. 2020 Dai et al. This content is distributed under the terms of the Creative Commons Attribution 4.0 International license.

We found that the percentage of phagocytosis of RBCs from DAT-positive patients with HIV was significantly higher than the percentage seen with RBCs from healthy donors and DAT-negative patients with HIV (Fig. 1B and C). No significant differences in erythrophagocytosis were observed between DAT-negative patients with HIV and healthy donors (Fig. 1B and C). TB status did not influence the differences in erythrophagocytosis between HIV patients with TB and those without TB, which might have been due to the limited number of cases (*n* = 5) of HIV-TB (see Fig. S1 in the supplemental material). The DAT reactions were classified in ascending degrees from negative (-) to positive, with 1+, 2+, and 3+ corresponding to the levels of agglutination strength of RBCs (Fig. 1D). We found that the extent of erythrophagocytosis was significantly correlated with the agglutination degree of DAT (*R* = 0.99 and *P* < 0.01 for both) (Fig. 1E), indicating the importance of autoantibodies in erythrophagocytosis.

10.1128/mBio.03246-19.1 FIG S1 TB status has no effect on macrophage-mediated phagocytosis of RBCs from patients with HIV. Data represent *ex vivo* phagocytosis of PKH-26-labeled RBCs from healthy donors (*n* = 33) and HIV patients with TB (*n* = 5) or without TB (*n* = 42) upon incubation with PMA-differentiated THP-1 macrophages for 6 h at RBC/macrophage (R/M) ratios of 10:1 and 100:1, respectively. Download FIG S1, TIF file, 1.1 MB. Copyright © 2020 Dai et al. 2020 Dai et al. This content is distributed under the terms of the Creative Commons Attribution 4.0 International license.

**FIG 2:**
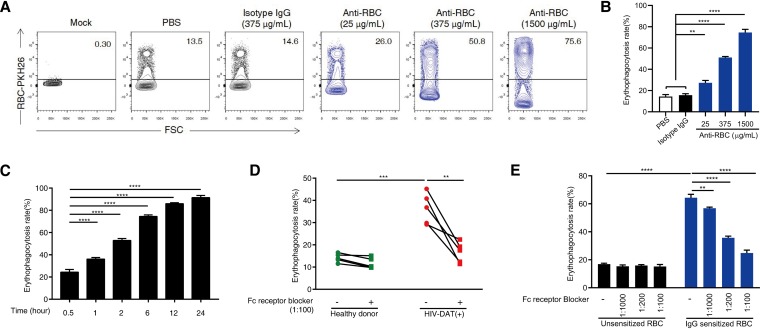
Enhanced erythrophagocytosis in patients with HIV can be blocked by neutralizing Fcγ receptors on macrophages. (A and B) RBCs from healthy donors were sensitized with different concentrations of anti-human RBC IgG (0, 25, 375, and 1,500 μg/ml) or isotype rabbit IgG (375 μg/ml) and were then labeled with PKH-26. *In vitro* phagocytosis of the indicated RBCs after incubation with PMA-differentiated THP-1 macrophages for 6 h at an RBC/macrophage (R/M) ratio of 100:1 was revealed by forward scatter data (A) and quantified by flow cytometry (B). (C) Erythrophagocytosis analysis was performed at various time points (0.5, 1, 2, 6, 12, and 24 h) after treatment of sensitized RBCs (sRBCs) (anti-human RBC IgG, 1,500 μg/ml) at an R/M ratio of 100:1, as assessed by flow cytometry. (D and E) PMA-differentiated THP-1 macrophages were treated with the indicated dilution ratios of Fcγ receptor blocker for 2 h followed by exposure to PKH-26-labeled RBCs from healthy donors (*n* = 5) or from patients with HIV with a positive direct antiglobulin test (DAT) result (*n* = 5) or to sRBCs for another 6 h. Healthy RBCs without sensitization were used as a control. The rate of erythrophagocytosis was quantified by flow cytometry. The data represent means ± standard deviations and are representative of results from at least three independent experiments. **, *P* < 0.01; ***, *P < *0.001; ****, *P < *0.0001.

We further demonstrated a role for anti-RBC autoantibodies in erythrophagocytosis *in vitro* by preincubating RBCs from healthy donors with commercial anti-human RBC IgG to mimic RBC sensitization *in vivo*. These sensitized RBCs (sRBCs) were more susceptible to erythrophagocytosis than nonsensitized RBCs incubated with vehicle (phosphate-buffered saline [PBS]) or isotype rabbit IgG (Fig. 2A and B). This increase in erythrophagocytosis was both anti-RBC IgG dose dependent and time dependent (Fig. 2B and C). We then used Fcγ receptor blocking antibodies to block the Fcγ receptors on macrophages that sense antibody-RBC immunocomplexes and mediate RBC phagocytosis. Here, we found that the erythrophagocytosis of RBCs purified from DAT-positive patients with HIV and sRBCs by anti-RBC IgG was completely inhibited, whereas erythrophagocytosis of RBCs from healthy donors was not affected by Fcγ receptor blocker treatment (Fig. 2D and E). In contrast, the complement receptor was not involved in erythrophagocytosis (Fig. S2). Taken together, these results demonstrate that enhanced erythrophagocytosis in DAT-positive patients with HIV is mediated by anti-RBC autoantibodies. In addition, the data suggest that sRBCs constitute a reliable model to further study erythrophagocytosis.

10.1128/mBio.03246-19.2 FIG S2 The C1qR complement receptor is not involved in autoantibody-mediated erythrophagocytosis. (A and B) PMA-differentiated THP-1 macrophages were treated with the indicated dilution ratios of anti-C1q receptor antibody and Fcγ receptor blocker for 2 h followed by incubation with PKH-26-labeled sRBCs for another 6 h as indicated (A), and the rate of erythrophagocytosis was quantified by flow cytometry (B). FSC, forward scatter. ****, *P < *0.0001. Download FIG S2, TIF file, 1.9 MB. Copyright © 2020 Dai et al. 2020 Dai et al. This content is distributed under the terms of the Creative Commons Attribution 4.0 International license.

### Enhanced erythrophagocytosis impairs macrophage bactericidal activity against M. tuberculosis by inhibiting autophagy.

**FIG 3:**
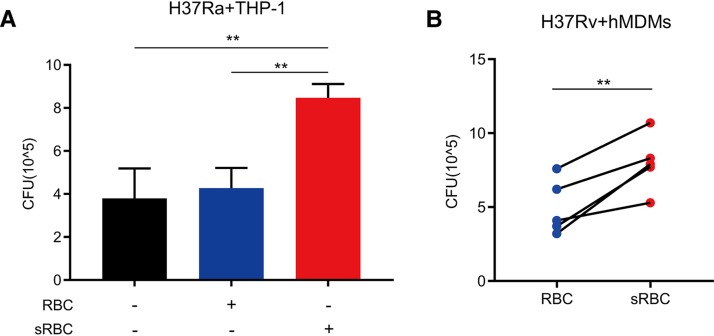
Enhanced erythrophagocytosis increases intracellular Mycobacterium tuberculosis growth. (A) PMA-differentiated THP-1 macrophages were infected with H37Ra (MOI = 10:1) for 6 h. The cells were then treated with unsensitized RBCs or sensitized RBCs (sRBCs) (anti-human RBC IgG, 1,500 μg/ml) at an R/M ratio of 100:1 for 72 h. Macrophage bactericidal activity was assessed by CFU counts of intracellular H37Ra. The data represent means ± standard deviations and are representative of results from at least three independent experiments. (B) Adherent monocytes enriched from PBMCs (from five healthy donors) were allowed to differentiate into human monocyte-derived macrophages (hMDMs) under conditions of stimulation (50 ng/ml macrophage colony-stimulating factor [M-CSF]) in culture. Intracellular CFU levels in hMDMs were assessed for live virulent M. tuberculosis as described for panel A. **, *P < *0.01.

Our findings that anti-RBC autoantibodies are an independent risk factor for TB in patients with HIV and that they have a direct effect on erythrophagocytosis prompted us to investigate whether and how erythrophagocytosis influences macrophage-mediated immunity against M. tuberculosis infection. We found that erythrophagocytosis significantly impaired the bactericidal activity of PMA-differentiated THP-1 macrophages against M. tuberculosis H37Ra (Fig. 3A). Specifically, compared to unsensitized RBC treatment and controls, treatment with sRBCs significantly increased intracellular H37Ra growth (Fig. 3A). Similar results were obtained when the virulent M. tuberculosis H37Rv strain was used to infect primary human monocyte-derived macrophages (hMDMs) (Fig. 3B).

**FIG 4:**
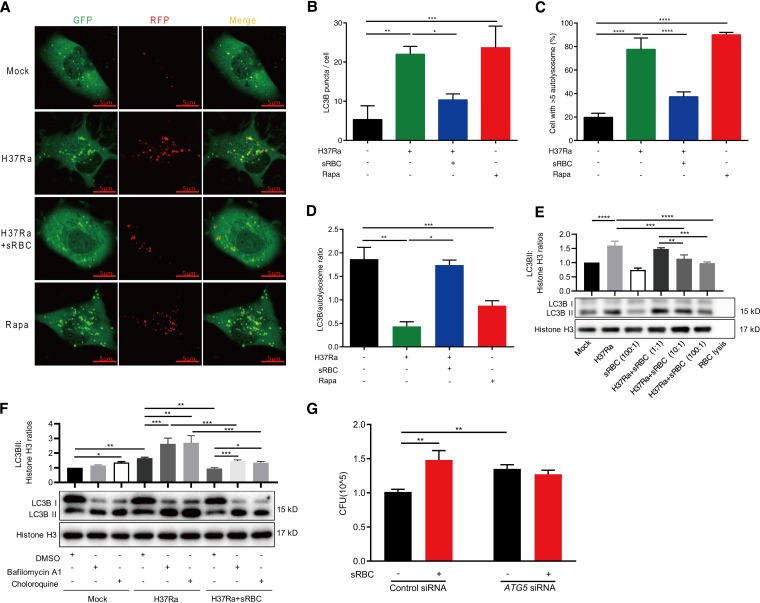
Enhanced erythrophagocytosis impairs macrophage bactericidal activity against Mycobacterium tuberculosis by inhibiting autophagy. (A to D) mRFP-GFP-LC3B reporter THP-1 cells were differentiated by the use of PMA and infected with H37Ra (MOI = 10:1) in the presence or absence of sensitized RBCs (sRBCs) (anti-human RBC IgG, 1,500 μg/ml; R/M ratio = 100:1) for 24 h. Cells treated with 10 nM rapamycin were used as a positive control. (A) Representative confocal microscopy images are shown (bars, 5 μm). (B to D) The number of LC3 puncta (green)/cell (B), the percentage of cells with >5 autolysosomes (red) (C), and the LC3/autolysosome ratio (D) were calculated. (E) PMA-differentiated THP-1 macrophages were infected with H37Ra (MOI = 10:1) for 24 h in the presence or absence of sRBCs at the indicated RBC/macrophage ratios. The lysates from the panel of cells were prepared as described for panel A, and only RBC lysates were analyzed by Western blotting. LC3B-II/histone H3 ratios shown above. (F) PMA-differentiated THP-1 macrophages were infected with H37Ra (MOI = 10:1) for 24 h in the presence or absence of sRBCs (100:1), with or without bafilomycin A1 (100 nM) and chloroquine (25 μM) treatment for another 3 h. The lysates from the panel were analyzed by Western blotting. LC3B-II/histone H3 ratios are shown at the top. DMSO, dimethyl sulfoxide. (G) PMA-differentiated THP-1 macrophages were transfected with *ATG5* small interfering RNA (siRNA). Scrambled siRNA was used as a negative control. After 36 h, the cells were infected with H37Ra (MOI = 10:1) for 6 h and then treated with sRBCs (R/M ratio = 100:1) for a further 72 h. The panel of cells was lysed in 0.1% SDS and plated on 7H10 plates for calculating CFU. The data represent means ± standard deviations and are representative of results from at least three independent experiments. *, *P < *0.05, **, *P < *0.01; ***, *P < *0.001; ****, *P < *0.0001.

We then explored the mechanisms by which erythrophagocytosis enhances M. tuberculosis intracellular survival. Because heme/iron metabolism and homeostasis following erythrophagocytosis require active phagolysosome biogenesis, involving the interactions of nascent phagosomes with endocytic and autophagy compartments (24), we first investigated whether phagosome maturation is affected by sRBCs. The results showed that the pH value of phagosome did not significantly change with or without sRBC treatment in H37Ra-infected macrophages (Fig. S3), suggesting that phagosome maturation is not affected by sRBC treatment. We then focused our study on the role of autophagy in erythrophagocytosis-mediated impairment in bacterial control. We used THP-1 cells transfected with the mRFP-GFP-LC3B (monomeric red fluorescent protein-green fluorescent protein-LC3B) reporter (25), a gold standard system for measuring autophagy and monitoring the rate of autophagic flux and the maturation of autophagosomes simultaneously, to evaluate the effect of erythrophagocytosis on autophagy (26). Using this system, when autophagosomes mature and fuse with lysosomes to form autolysosomes, GFP degrades in the acidic environment of autolysosomes, whereas mRFP is stable. Therefore, the presence of yellow puncta indicates autophagosome formation (or autophagic flux), and the presence of red puncta indicates autophagosome maturation and the formation of autolysosomes (27). Infection with H37Ra and treatment with the autophagy inducer rapamycin induced almost equal levels of autophagosome formation and maturation; conversely, treatment with sRBCs led to a reduction in autophagic flux (Fig. 4A to D). This result was confirmed by Western blot analysis of the lipidated and autophagosome-associated LC3B-II form of LC3B (Fig. 4E). We further used chloroquine and bafilomycin A1, potent inhibitors of autophagosome-lysosome fusion and acidification, to determine whether the reduction of LC3B-II in the sRBC treatment group was due to an increase in autophagy flux. As expected, both chloroquine and bafilomycin A1 promoted the accumulation of LC3B-II induced by M. tuberculosis infection (Fig. 4F). These inhibitors did not further increase LC3B-II accumulation in the presence of sRBCs, suggesting that sRBC treatment inhibited autophagy in M. tuberculosis-infected macrophages (Fig. 4F).

10.1128/mBio.03246-19.3 FIG S3 Phagosome maturation of H37Ra-infected macrophages is unaffected by sRBC treatment. (A and B) PMA-differentiated THP-1 macrophages were infected with H37Ra (MOI = 10:1) in the presence or absence of sRBCs (R/M ratio = 100:1) for 24 h. pHrodo Red BioParticles were used to detect the pH within phagosomes by flow cytometry. Download FIG S3, TIF file, 1.3 MB. Copyright © 2020 Dai et al. 2020 Dai et al. This content is distributed under the terms of the Creative Commons Attribution 4.0 International license.

To further confirm the inhibition of autophagy by erythrophagocytosis in intracellular M. tuberculosis growth, we used small interfering RNA (siRNA) to silence a key autophagy-related gene, *ATG5*, to interfere with autophagy progression. *ATG5* siRNA transfection markedly decreased *ATG5* gene expression but had no effect on RBC and M. tuberculosis phagocytosis by macrophages (Fig. S4). Consistent with a previous report (28), we observed that *ATG5* knockdown increased the survival of intracellular M. tuberculosis; however, unlike control siRNA treatment, the intracellular M. tuberculosis CFU count in the *ATG5* knockdown macrophages did not increase upon sRBC exposure (Fig. 4G). These results indicate that enhanced erythrophagocytosis impairs macrophage bactericidal activity by inhibiting autophagy.

10.1128/mBio.03246-19.4 FIG S4 *ATG5* and *HMOX-1* knockdown has no effect on macrophage-mediated phagocytosis of sensitized RBCs and M. tuberculosis. PMA-differentiated THP-1 macrophages were transfected with *ATG5* and *HMOX-1* siRNA (50 nM). Scrambled siRNA was used as a negative control. (A) Relative expression levels of *ATG5* were detected by quantitative PCR (qPCR). (B and C) *In vitro* phagocytosis of PKH-26-labeled RBCs after incubation for 6 h with silenced THP-1 macrophages at an RBC/macrophage ratio of 100:1. Data shown were quantified by flow cytometry. (D) *ATG5* and *HMOX-1* knockdown THP-1 macrophages were infected with H37Ra (MOI = 10:1) for 6 h before calculating levels of intracellular CFUs. The data represent means ± standard deviations and are representative of results from at least three independent experiments. **, *P < *0.01. Download FIG S4, TIF file, 2.6 MB. Copyright © 2020 Dai et al. 2020 Dai et al. This content is distributed under the terms of the Creative Commons Attribution 4.0 International license.

### Erythrophagocytosis inhibits macrophage-mediated autophagy via heme oxygenase-1 (HO-1) expression.

**FIG 5:**
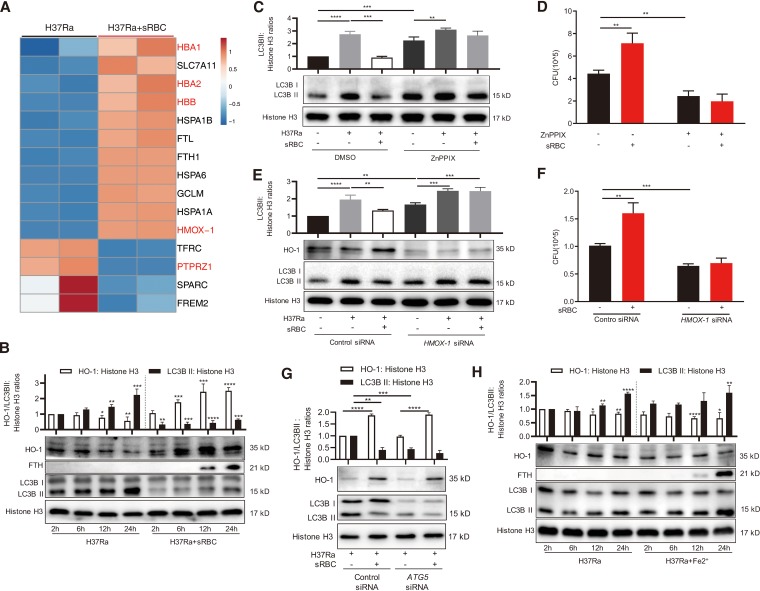
Erythrophagocytosis inhibits macrophage autophagy via regulating heme oxygenase-1 (HO-1) expression. (A) PMA-differentiated THP-1 macrophages were infected with H37Ra (MOI = 10:1) in the presence or absence of sensitized RBCs (sRBCs) (anti-human RBC IgG, 1,500 μg/ml; R/M ratio = 100:1) for 2, 6, 12, and 24 h. Total RNA was extracted for library construction and sequencing. The heat map shows the top 15 differentially expressed genes (DEGs) (selected on the basis of an adjusted *P* value of *<*0.05 and fold changes of FPKM) in comparisons of H37Ra-infected macrophages treated with or without sRBCs for 12 h. Blue, low expression; red, high expression. The genes differentially expressed at all time points (2, 6, 12, and 24 h) are marked in red. (B) PMA-differentiated THP-1 macrophages were infected with H37Ra (MOI = 10:1) in the presence or absence of sRBCs (R/M ratio = 100:1) for the indicated times. HO-1, ferritin heavy chain (FTH), and LC3B protein levels were examined by Western blotting. (C and E) Expression of HO-1 was monitored in macrophages exposed to the pharmacological inhibitor ZnPPIX (2.5 μM) or DMSO (C) or to specific control siRNA or *HMOX-1* siRNA (50 nM) (E) after infection with H37Ra in the presence or absence of sRBCs for 24 h. LC3B protein levels were examined by Western blotting. (D and F) Intracellular H37Ra CFU levels were assessed in macrophages prepared as described for panels C and E followed by sRBC treatment for 72 h. (G) TPH-1 macrophages with *ATG5* knockdown were infected with H37Ra (MOI = 10:1) in the presence or absence of sRBCs (R/M ratio = 100:1) for 24 h. HO-1 and LC3B protein levels were examined by Western blotting. (H) PMA-differentiated THP-1 macrophages were infected with H37Ra (MOI = 10:1) in the presence or absence of ferrous lactate (200 μM) for the indicated times. HO-1, FTH, and LC3B protein levels were examined by Western blotting. HO-1 and LC3B-II/histone H3 ratios are shown at the top. The data represent means ± standard deviations and are representative of results from at least three independent experiments. *, *P < *0.05; **, *P < *0.01; ***, *P < *0.001; ****, *P < *0.0001.

Increased RBC damage or intravascular hemolysis can lead to acute heme accumulation in macrophages (29). In response, macrophages promptly upregulate a number of protective mechanisms: these protective responses involve heme catabolism by HO-1 and iron sequestration by ferritin (29, 30). We thus performed RNA sequencing (RNA-seq) to profile the transcriptomes of macrophages infected with H37Ra in the absence or presence of sRBCs. We found that genes directly associated with heme degradation were expressed at higher levels in H37Ra-infected macrophages treated with sRBCs for 12 h than i those without sRBC exposure, including ferritin light chain (*FTL*), ferritin heavy chain 1 (*FTH1*), hemoglobin subunit alpha 1/2 (*HBA1*/*2*), hemoglobin subunit beta (*HBB*), and heme oxygenase-1 (*HMOX-1*) (Fig. 5A; see also Fig. S5). Among them, *HMOX-1* was differentially expressed at all indicated time points, including 2, 6, 12, and 24 h (Fig. S5). We confirmed a time-dependent expression pattern for HO-1 and FTH by Western blotting and found that the increased expression coincided with the reduced LC3B-II expression (Fig. 5B).

10.1128/mBio.03246-19.5 FIG S5 Differentially expressed genes (DEGs) from comparisons between H37Ra-infected THP-1 cells treated with or without sensitized RBCs. PMA-differentiated THP-1 macrophages were infected with H37Ra (MOI = 10:1) in the presence or absence of sensitized RBCs (sRBCs) (anti-human RBC IgG was used at 1,500 μg/ml; R/M ratio = 100:1) for 2, 6, 12, and 24 h. Total RNA was extracted for library construction and sequencing. (A) The top 15 DEGs from comparisons between H37Ra-infected macrophages treated with or without sRBCs for 12 h. (B to P) The expression level of the 15 genes referred to above at different time points (2, 6, 12, and 24 h) in H37Ra-infected macrophages treated with or without sRBCs (red indicates genes identified in the presence of sRBCs). (Q) Venn diagram of the DEGs identified by RNA sequencing in H37Ra-infected macrophages treated with or without sRBCs. Download FIG S5, TIF file, 2.8 MB. Copyright © 2020 Dai et al. 2020 Dai et al. This content is distributed under the terms of the Creative Commons Attribution 4.0 International license.

As HO-1 regulates autophagy (31, 32), we hypothesized that erythrophagocytosis might inhibit autophagy by inducing HO-1 expression. To test this hypothesis, we infected PMA-differentiated THP-1 macrophages with H37Ra and then exposed them to the HO-1 inhibitor zinc protoporphyrin (ZnPPIX; 2.5 μM) and to sRBCs for 24 h. Western blot analysis showed that ZnPPIX treatment partly relieved the inhibitory effect of erythrophagocytosis on LC3B-II expression (Fig. 5C). Importantly, we found that ZnPPIX exposure inhibited intracellular bacterial growth compared with the noninhibitor control group; this defect was not rescued by sRBC treatment (Fig. 5D).

Exposure to HO-1 inhibitors leads to cellular toxicity due to the production of reactive oxygen species (ROS) in the specific context of photochemical activation (27, 33). Treating H37Ra-infected macrophages with ZnPPIX, however, did not increase cytosolic reactive oxygen species (cROS) production. Rather, it actually decreased the cROS level (Fig. S6). To exclude the possibility of direct bactericidal activity (data not shown) and cellular toxicity induced by ZnPPIX, we therefore used *HMOX-1* siRNA to specifically verify the role of HO-1 in inhibiting autophagy during erythrophagocytosis. Transfection with *HMOX-1* siRNA markedly decreased the HO-1 protein level in macrophages (Fig. 5E) without affecting sRBC and M. tuberculosis phagocytosis (Fig. S4) and cell deaths (Fig. S7). Consistent with the effects of ZnPPIX, we found that *HMOX-1* knockdown in H37Ra-infected macrophages significantly abrogated the inhibitory effect of erythrophagocytosis on the expression of LC3B-II (Fig. 5E). In contrast, impaired autophagy driven by *ATG5* silencing had no effect on HO-1 expression (Fig. 5G). Most importantly, in accordance with our chemical inhibitor results (Fig. 5E) and with a previous report (34), *HMOX-1* knockdown completely eradicated the inhibitory effects of erythrophagocytosis on the macrophage defense against M. tuberculosis, as determined by the CFU count of intracellular M. tuberculosis (Fig. 5F). Taken together, these findings confirm that the increased level of intracellular M. tuberculosis survival in response to erythrophagocytosis depends on the inhibition of HO-1-regulated autophagy.

10.1128/mBio.03246-19.6 FIG S6 ZnPPIX treatment decreases cROS production in M. tuberculosis-infected macrophages. (A and B) PMA-differentiated THP-1 macrophages were infected with H37Ra (MOI = 10:1) in the presence (A) or absence (B) of ZnPPIX (2.5 μM). cROS production was determined by flow cytometry. ****, *P < *0.0001. Download FIG S6, TIF file, 0.7 MB. Copyright © 2020 Dai et al. 2020 Dai et al. This content is distributed under the terms of the Creative Commons Attribution 4.0 International license.

10.1128/mBio.03246-19.7 FIG S7 *HMOX-1* knockdown has no effect on cell death of macrophages infected with M. tuberculosis. PMA-differentiated THP-1 macrophages were transfected with *HMOX-1* siRNA (50 nM). Scrambled siRNA was used as a negative control. (A and B) Cell deaths were determined using an annexin V/propidium iodide (PI) kit after H37Ra infection (MOI = 10:1) for 24 h by flow cytometry. Download FIG S7, TIF file, 1.6 MB. Copyright © 2020 Dai et al. 2020 Dai et al. This content is distributed under the terms of the Creative Commons Attribution 4.0 International license.

As previously described (35), erythrophagocytosis also leads to an increase in iron levels, which might benefit intracellular M. tuberculosis growth by compensatory iron acquisition. In our final assays, we found that treating H37Ra-infected macrophages with ferrous lactate (200 μM) neither increased HO-1 levels nor suppressed LC3B-II levels (Fig. 5H). As blocking HO-1 completely reversed the inhibitory effects caused by erythrophagocytosis, we reason that erythrophagocytosis supports M. tuberculosis intracellular survival by inhibiting autophagy rather than depending fully on heme-iron acquisition.

## DISCUSSION

CD4 T-cell depletion has long been considered an important risk factor for TB in HIV. Although the details of the mechanism remain uncertain, recent data have emerged to show that HIV infection may compromise macrophage function to impair host control of M. tuberculosis (2, 10, 36, 37). To the best of our knowledge, we report for the first time that increased TB susceptibility is significantly associated with anti-RBC autoantibody production (as determined by DAT) in patients with HIV. Enhanced erythrophagocytosis of RBCs by macrophages in DAT-positive patients significantly correlated with anti-RBC autoantibody production and was efficiently blocked with inhibition of Fcγ receptors rather than complement receptors. The erythrophagocytosis of RBCs sensitized with anti-human RBCs IgG induced excessive expression of HO-1, which inhibited autophagy and impaired macrophage bactericidal activity against M. tuberculosis. These findings provide novel insights into how HIV infection increases TB susceptibility by impairing macrophage anti-TB immunity.

HIV infection induces autoantibody production via mechanisms such as (i) direct B-cell activation as a result of antigen similarity and (ii) dysregulated B-cell activation due to CD4 T-cell depletion (14, 38). Anti-RBC autoantibodies, which sensitize RBCs for destruction, have also been implicated in HIV-associated autoimmune hemolytic anemia (39, 40). A positive DAT result and anemia are frequently observed in the clinical syndrome of HIV infection, and these two parameters are significantly associated with each other (16, 22). Additionally, the severity of anemia can be used to predict the incidence of and mortality associated with TB in patients with HIV (21). Consistently, our univariate analysis showed that the rate of anemia was significantly associated with TB incidence in patients with HIV; however, this association was lost in a multivariate analysis. While we saw no significant association between TB incidence and the number of CD4 T cells—possibly because all HIV patients recruited to this study were at an early clinical stage and still had a relatively high level of CD4 T cells—we discovered a strong association between a positive DAT result and the incidence of TB. As the antiviral drugs used for HIV treatment have the potential to induce drug-associated RBC hemolysis and present false-positive DAT results (20), we recruited only treatment-naive patients with HIV in the present study to avoid the potentially confounding factor of drug use. Taken together, our data show that anti-RBC autoantibody production is an independent risk factor for TB susceptibility in patients with HIV, regardless of the presence of anemia.

M. tuberculosis has evolved a highly efficient iron-acquiring system to compete for iron, particularly within extremely hostile environments such as within macrophages (35, 41, 42). Although we cannot entirely rule out the contribution of heme as an iron source for increased intracellular M. tuberculosis growth caused by enhanced erythrophagocytosis, disruption of the autophagic response, by targeting either HO-1 or *ATG5*, completely abrogated the effects of erythrophagocytosis on macrophagic bactericidal activity against M. tuberculosis. In addition, treatment with iron (ferrous lactate) did not increase the expression of HO-1 or inhibit autophagy in M. tuberculosis-infected macrophages. Together, these data suggest that the contribution of iron to increased intracellular M. tuberculosis growth during erythrophagocytosis is limited. Further investigations using an M. tuberculosis mutant with deficient heme iron uptake systems may provide direct evidence as to whether iron from RBCs serves as a nutrient that is beneficial to intracellular M. tuberculosis.

HO-1, encoded by *HMOX-1*, is an inducible cytoprotective enzyme that catalyzes the oxidative degradation of heme into equimolar ratios of iron, carbon monoxide, and bilirubin (43). Although the transcript, protein, and activity levels of HO-1 are upregulated in response to M. tuberculosis infection, whether HO-1 has a protective or pathogenic role in TB remains controversial (17, 34, 44–47). In support of our findings, several reports have shown that pharmacological HO-1 inhibition decreases M. tuberculosis and Mycobacterium abscessus burden *in vivo* (44) and *in vitro* (34, 45). Conversely, others have shown that HO-1 is required to control M. tuberculosis and Mycobacterium avium infections in mice (17, 46, 47). These conflicting findings might be due to the off-target effects of HO-1 chemical inhibitors and to the fact that the essentiality of HO-1 in mice and humans varies; however, it should be highlighted that the roles of HO-1 in restricting M. tuberculosis infection may diverge under different circumstances in a manner that depends on the dynamics of HO-1 expression. During normal RBC homeostasis, the expression and enzyme activity of HO-1 is induced but is tightly regulated to counteract oxidative stress or heme, with the purpose to protect macrophages from death, and therefore benefits the host defense against TB. However, under circumstances such as enhanced erythrophagocytosis in patients with HIV, expression of excessive HO-1 is induced and sustained, which inhibits autophagy and thus facilitates intracellular M. tuberculosis growth (47). In agreement with previous reports (48), we found that HO-1 was significantly upregulated in macrophages at an early time point (∼6 h) after M. tuberculosis infection. HO-1 expression, however, decreased at a late time point (∼12 h) following infection. Notably, erythrophagocytosis not only significantly increased the M. tuberculosis infection-induced upregulation of HO-1 but also sustained its expression up to 24 h after infection.

A similar phenomenon is the role of HO-1 in the modulation of autophagy. Depending on the cell type and stressor, HO-1 can inhibit or induce autophagy in a signal-specific and cell line-specific manner. For example, induction of autophagy is dependent on HO-1 in primary mouse macrophages and hepatocytes (49, 50). In lung and colon cancer, HO-1-mediated autophagy sustains cancer cell survival and leads to a more aggressive phenotype (51, 52). Conversely, recent studies have shown that HO-1 may also inhibit autophagy to protect renal tubules from cisplatin-mediated injury (31) and modulate hyperthermia-induced antiviral effects in cervical cancer cells (32). We believe, therefore, that it is important to further address the reasons underlying the paradoxical effects of HO-1 on control of M. tuberculosis infection and regulating autophagy. Nevertheless, our study results confirm that HO-1 induction by anti-RBC autoantibody-mediated erythrophagocytosis is essential and sufficient to impair macrophage bactericidal activity against M. tuberculosis by inhibiting *ATG5*-dependent autophagy by applying pharmacological inhibitors of HO-1 and knocking down HO-1 with siRNA.

In summary, we have shown that HIV infection increases autoantibody-mediated erythrophagocytosis, which impairs macrophagic bactericidal activity against M. tuberculosis by inhibiting HO-1-associated autophagy. These findings reveal a novel mechanism by which HIV infection increases susceptibility to TB and provide evidence that DAT could be a potential measure to identify HIV patients who are at high risk of TB and who thus would be suitable candidates for anti-TB chemotherapy preventive treatment.

## MATERIALS AND METHODS

### Ethics statement.

The present study was approved by the Ethics Committees of the Shenzhen Third Hospital (Shenzhen, China). Written informed consent was provided by all study participants.

### Study population and samples.

To investigate the presence of red blood cell (RBC) autoantibodies in patients with HIV and its association with the susceptibility to tuberculosis (TB), 281 HIV-seropositive patients were recruited from the Shenzhen Third Hospital (Shenzhen, China) between August 2017 and November 2018. All participants underwent clinical assessment at recruitment, and information was collected on age, sex, HIV copy numbers, hemoglobin level, white blood cell (WBC) count, CD4 T-cell count, direct antiglobulin test (DAT) result, and results from examinations conducted for diagnosing TB, such as chest radiography, interferon gamma release assay (IGRA), and sputum microbiological tests, including acid-fast bacillus (AFB) staining, nucleic acid amplification test (NAAT), and Mycobacterium tuberculosis culture. Among the potential subjects, 14 cases were excluded due to missing data or questionable diagnosis. Subsequently, a total of 267 cases that had a conclusive clinical diagnosis were included in the study. The clinical and demographic data are shown in Table 1. As described previously (53), TB was diagnosed by experienced specialists if (i) sputum samples were positive for AFB, NAAT, or M. tuberculosis culture or (ii) the results showed no sputum evidence or a negative smear but did show high-resolution computed tomography evidence, positive IGRA, and symptoms responding to TB treatment.

For erythrophagocytosis analysis, 33 healthy donors and 47 HIV-seropositive patients who were not coinfected with TB (including 36 DAT-negative cases and 11 DAT-positive cases) were selected (see Table S1 in the supplemental material). Here, 5 ml whole blood was collected for RBC and human peripheral blood mononuclear cell (PBMC) isolation and 5 ml for DAT by venipuncture, using heparinized tubes and ethylenediaminetetraacetic acid (EDTA) tubes (Sigma), respectively.

### Cell isolation and culture.

Human monocytic THP-1 (TIB-202; ATCC) cells and THP-1 cells transformed with an mRFP-GFP-LC3B reporter (provided by Hongbo Shen, Institute Pasteur of Shanghai, Shanghai, China) were grown in RPMI 1640 medium supplemented with l-glutamine (2 mM) and 10% heat-inactivated fetal bovine serum (FBS) (all from Gibco, Life Technologies). As previously described (54), the THP-1 cells were plated at 4 × 10^5^ cells/ml in RPMI 1640 (10% FBS, 2 mM l-glutamine) and treated with 40 ng/ml phorbol 12-myristate 13-acetate (PMA; Sigma-Aldrich) in 12-well or 6-well plates (Costar) for 48 h and allowed to differentiate into macrophages, unless otherwise indicated. The cells were then incubated with fresh prewarmed RPMI 1640 (10% FBS, 2 mM l-glutamine) and maintained in the media at 37°C until further use.

PBMCs were isolated by density gradient centrifugation (400 × *g*, 30 min) using Ficoll-Paque medium (GE), as previously described (55). The PBMCs were then incubated overnight to enrich for monocytes by adherence on plastic culture plates. Nonadherent cells were removed via two washes with prewarmed RPMI 1640, and the adherent monocytes were treated with 50 ng/ml human macrophage colony stimulating factor (PeproTech)–RPMI 1640 (supplemented with 10% FBS and 2 mM l-glutamine) for 5 days to enable differentiation into human monocyte-derived macrophages (hMDMs).

Human fresh RBCs from whole blood were separated by density gradient centrifugation (400 × *g*, 30 min). Following centrifugation, all layers on RBCs were carefully removed. The sedimented RBCs were washed three times with phosphate-buffered saline (PBS) and then underwent WBC depletion by filtration (Pall) (56). The RBCs isolated from patients with HIV and healthy donors were stored in citrate phosphate dextrose buffer (Baxter Healthcare) at 4°C for use within 3 days.

### DAT.

The DAT was run using a Grifols DG gel system with Coombs cards and monospecific reagent (all from Grifols Diagnostic Solutions Inc.) to detect bound IgG. The whole blood was anticoagulated with EDTA and then centrifuged (400 × *g*, 5 min) to separate the cells from the plasma. The sedimented RBCs were washed three times and resuspended in PBS to a final concentration of 1%. Following this, 50 μl 1% RBCs was added to microtubes and centrifuged for 9 min using a preset cycle for antibody identification. The results of reactions were generated automatically through artificial vision technology and evaluated using a scale of strength as follows: negative (-) or increasingly positive at 1+, 2+, 3+, or 4+ (20, 57). The experiment was performed under the supervision of trained and experienced medical technicians.

### RBC preparation and erythrophagocytosis assay.

The isolated RBCs were washed three times and then labeled with a membrane-integrating dye (PKH-26) (Sigma), according to the manufacturer’s instructions (23). In detail, a 2× cell suspension containing 5 × 10^7^ RBCs in 1 ml diluent C buffer was prepared and then rapidly mixed with dye solution (2×) containing 4 μl PKH-26 dye in 1 ml diluent C buffer. After incubation for 5 min at room temperature, an equal volume (2 ml) of FBS (Gibco, Life Technologies) was added to stop the staining. The labeled RBCs were washed three times and resuspended in RPMI 1640 for the erythrophagocytosis assay. Prior to labeling, the RBCs were sensitized (if required) by incubating the cells in 25, 375, and 1,500 μg/ml commercial rabbit anti-human RBC IgG (Aviva Systems Biology) or isotype rabbit IgG (Abcam) at room temperature for 30 min and then washed three times and resuspended in diluent C buffer for labeling.

For the erythrophagocytosis assay, the PMA-differentiated THP-1 macrophages were pretreated with or without Fcγ receptor blocker (containing anti-CD16, anti-CD32, and anti-CD64; BioLegend) and anti-C1q receptor antibody (ImmunoWay Biotechnology Company) at dilution ratios of 1:1,000, 1:200, and 1:100 for 2 h and were then treated with PKH-26-labeled RBCs at the indicated RBC/macrophage (R/M) ratios of 10:1 and 100:1 for 0.5, 1, 2, 6, 12, and 24 h. The culture supernatants were then removed, and 1 ml RBC lysis buffer (Solarbio) was added to each well followed by incubation for 5 min at room temperature. The cells were washed once in PBS and collected following trypsin (Gibco, Life Technologies) digestion for 5 min. The macrophage erythrophagocytosis rate was analyzed using flow cytometry (BD FACSAria II) and FlowJo software version 10 (BD).

### M. tuberculosis culture, cell infection, and measurement of CFU.

M. tuberculosis virulent H37Rv and attenuated H37Ra strains were both cultured in Middlebrook 7H9 medium supplemented with 10% oleic acid-albumin-dextrose-catalase (OADC) enrichment medium (Becton, Dickinson), 0.2% (vol/vol) glycerol (Sigma), and 0.25% (vol/vol) Tween 80 (Sigma) at 37°C, as previously described (58). M. tuberculosis in mid-log-phase growth was used for infection experiments at an optical density (OD) of 0.6. The OD was correlated with the CFU level after colony counts were performed in triplicate on Middlebrook 7H10 agar supplemented with 10% OADC, 0.5% glycerol, and 1g/liter l-asparagine (Sigma).

The PMA-differentiated THP-1 macrophages and hMDMs were infected with M. tuberculosis (multiplicity of infection [MOI] = 10:1) for 6 h and then washed three times. The cells were treated with unsensitized RBCs or sensitized RBCs (sRBCs) (anti-human RBC IgG, 1,500 μg/ml; RBC/macrophage [R/M] ratio = 100:1) for a further 72 h. Macrophage bactericidal activity was assessed by determination of M. tuberculosis CFU counts obtained from the intracellular environments. The cells were lysed in 0.1% SDS, and lysates were plated at various dilutions on 7H10 plates, which were incubated at 37°C for 2 to 4 weeks before CFU counting was performed, as previously described (59).

### Confocal microscopy.

THP-1 cells (1 × 10^5^ cells/ml) transformed with the mRFP-GFP-LC3B reporter were differentiated by the use of PMA and infected with H37Ra (MOI = 10:1) in the presence or absence of sRBCs (R/M ratio = 100:1) for 24 h. A group treated with 10 nM rapamycin was used as a positive control. The cells were washed twice in PBS and fixed with 4% paraformaldehyde solution (Solarbio) for 15 min, and slides were prepared using antifade (Invitrogen), viewed under a confocal microscope (Nikon A1R), and processed using ImageJ software (NIH, USA). The level of autophagy was measured by enumerating the number of puncta per cell, and cells with >5 autolysosomes per 100 cells were counted.

### RNA interference and transfection.

*ATG5* and *HMOX-1* siRNA (RiboBio; siB12531154855-1-5 and siG0931881756-1-5, respectively) transfections were performed as previously described (60) using Lipofectamine RNAiMAX (Invitrogen) according to the manufacturer’s protocol. The siRNA and Lipofectamine complexes were prepared in Opti-MEM (Gibco) at a 1:1 ratio and added to the predifferentiated THP-1 macrophages for 24 h in a dropwise manner. The medium was replaced after 36 h, and scrambled siRNA was used as a negative control. HO-1 expression was also inhibited by 2.5 μM zinc protoporphyrin (Sigma) treatment. The efficiency of knockdown was determined in both cases by quantitative reverse transcription-PCR (RT-qPCR) and/or Western blotting.

### Western blotting.

Following performance of the treatments as described in a previous study (61), cells were washed with PBS and lysed on ice by incubation in radioimmunoprecipitation assay (RIPA) lysis buffer (Cell Signaling Technology) supplemented with a protease inhibitor cocktail (Roche). The protein concentration was estimated on a Qubit 4.0 Fluorometer using a Qubit protein assay kit (Thermo Fisher), and the samples were separated by SDS-PAGE and transferred onto a polyvinylidene difluoride membrane (Merck/Millipore). Following blocking with 5% skim milk (BD)–phosphate-buffered saline with Tween 20 (PBST) for 2 h at room temperature, the membranes were incubated with antibodies against LC3B (Sigma), HO-1, ferritin heavy chain, or histone H3 (obtained from Abcam) overnight at 4°C and were then incubated with peroxidase-conjugated secondary antibodies (Abcam) and visualized using ECL detection solution (Thermo Fisher). The digital images of the protein bands were acquired using an ImageQuant LAS 4000 system (GE Healthcare Bio-Science). The densitometry of the protein bands was analyzed using ImageJ software (NIH, USA).

### RNA-seq analysis.

Total RNA was isolated using total RNA kits (Omega) and subjected to cDNA library construction and RNA-seq by the Beijing Genomics Institute. In brief, mRNA sequencing was conducted using a BGISEQ-500 platform and the high-quality reads were aligned to the human reference genome (hg19) with Bowtie 2. The expression levels of each of the genes were normalized to the number of fragments per kilobase per million (FPKM) mapped reads in an exon model by RNA-seq expectation maximization (RSEM). Significantly differentially expressed genes were defined on the basis of a >2-fold expression difference versus the control with an adjusted *P* value of <0.05.

### Statistical analyses.

Analyses were performed using GraphPad Prism Version 6 (GraphPad Software, Inc.) and SAS Version 9.4 (SAS Institute, Inc.). Univariate and multivariate logistic regression models were used to assess the factors associated with TB susceptibility in patients with HIV. One-way analysis of variance (ANOVA) and Tukey’s correction test were used to analyze differences among multiple groups. An unpaired Student's *t* test was used to analyze differences between two groups. A paired Student's *t* test was used to analyze the differences in erythrophagocytosis rates between the macrophages incubated with and without Fcγ receptor blocker. A Pearson’s *t* test was used to analyze the correlation between the phagocytosis rates of RBCs from patients with HIV and the agglutination degrees from DAT. A *P* value of <0.05 was considered statistically significant.
