# Erratum for Dai et al., “Autoantibody-Mediated Erythrophagocytosis Increases Tuberculosis Susceptibility in HIV Patients”

**DOI:** 10.1128/mBio.00510-20

**Published:** 2020-03-31

**Authors:** Youchao Dai, Yi Cai, Xin Wang, Jialou Zhu, Xiaoqian Liu, Houming Liu, Linghua Li, Yinze Zhang, Shengze Liu, Zhihua Wen, Carl G. Feng, Xinchun Chen, Xiaoping Tang

**Affiliations:** a Research Institute of Infectious Diseases, Guangzhou Eighth People’s Hospital, Guangzhou Medical University, Guangzhou, China; b Guangdong Key Laboratory of Regional Immunity and Diseases, Department of Pathogen Biology, Shenzhen University School of Medicine, Shenzhen, China; c International Cancer Center, Shenzhen University General Hospital, Shenzhen University Clinical Medical Academy, Shenzhen, China; d Department of Infectious Disease, Shenzhen People’s Hospital, 2nd Clinical Medical College of Jinan University, Shenzhen, Guangdong Province, China; e Integrated Chinese and Western Medicine Postdoctoral Research Station, Jinan University, Guangzhou, Guangdong Province, China; f Clinical Laboratory, Shenzhen Third People’s Hospital, Shenzhen University School of Medicine, Shenzhen, China; g Yuebei Second People’s Hospital, Shaoguan, China; h Immunology and Host Defense Group, Department of Infectious Diseases and Immunology, Faculty of Medicine and Health, The University of Sydney, Sydney, NSW, Australia

## ERRATUM

Volume 11, no. 1, e03246-19, 2020, https://doi.org/10.1128/mBio.03246-19. The fifth author’s name was spelled incorrectly in the original article, published on 25 February 2020. This has been corrected as shown in this erratum.

