# Supplementary material for: Autoantibody-Mediated Erythrophagocytosis Increases Tuberculosis Susceptibility in HIV Patients
Source: mBio. 2020 Feb 25;11(1):e03246-19. doi: 10.1128/mBio.03246-19 (PMC7042700; doi:10.1128/mBio.03246-19)
Supplement: TABLE S1 [file mBio.03246-19-st001.doc]

|  | **Healthy donors**  **(n=33)** | **HIV with negative DAT**  **(n=36)** | **HIV with positive DAT**  **(n=11)** |
| --- | --- | --- | --- |
| Male sex (n, %) | 25/33 (76) | 28/36 (78) | 8/11 (73) |
| Age (years) | 39 (32-50) | 36 (29-42) | 39 (33-49) |
| HIV load (log) | NA | 4.70 (4.26-5.47) | 5.28 (4.47-5.31) |
| WBC count (WBC, 10^9^/l) | 7.10 (5.83-7.90) | 7.85 (6.30-8.40) | 9.00 (6.93-10.10) |
| Haemoglobin levels (g/l) | 146 (138-155) | 137 (129-139) | 126 (123-130) |
| Anemia (n, %)^a^ | 0/33 (0) | 7/36 (19) | 6/11 (55) |
| CD4 T-cell number (cells/μl) | 931 (857-973) | 346 (235-530) | 263 (204-338) |
| Coinfected with TB^b^ | NA | 3/36 (8.3) | 2/11 (18.2) |

Table S1 Demographics and clinical characteristics of the subjects selected for erythrophagocytosis assay

Definition of abbreviations: HIV = human immunodeficiency virus; DAT = direct antiglobulin test; WBC = white blood cell; NA = not applicable

Categorical variables are expressed as absolute numbers and percentage, whereas continuous variables are reported as the median and interquartile range.

^a^Anemia: hemoglobin level <130 g/l for males and <115 g/l for females.

^b^TB was diagnosed by experienced specialists if 1) sputum samples were positive for AFB, NAAT, or Mtb culture; 2) or no sputum or negative smear but high-resolution computed tomography (HRCT) evidence, positive IGRA, and symptoms responding to TB treatment.
